# Formulation and *in vitro* Evaluation of Alfuzosin Extended Release Tablets Using Directly Compressible Eudragit

**DOI:** 10.4103/0250-474X.56019

**Published:** 2009

**Authors:** M. A. Roni, G. Kibria, R. Jalil

**Affiliations:** Department of Pharmaceutical Technology, Faculty of Pharmacy, University of Dhaka, Dhaka-1000, Bangladesh

**Keywords:** Alfuzosin hydrochloride, Eudragit RS PO, hydroxypropylmethylcellulose, matrix tablet, release kinetics

## Abstract

The aim of the present study was the determination of formulation factors and the *in vitro* evaluation of an extended release dosage form of a freely soluble weakly basic drug (alfuzosin hydrochloride). Binary mixer of one hydrophilic polymer (hydroxypropylmethylcellulose) and one directly compressible Eudragit (RS PO) was used in tablets prepared by direct compression. The amounts of both polymers were taken as independent variables for the 3^2^ Factorial design. The percent drug releases at 1, 6, 12 and 20 h were selected as responses. The main effect and interaction terms were quantitatively evaluated using mathematical model. Dissolution data were fitted to zero order, first order, and Higuchi's release kinetics to evaluate kinetic data. Both the diffusion and erosion mechanisms were responsible for drug release as shown by the power law. The release of Alfuzosin was prolonged for 20 h by binary mixer indicating the usefulness of the formulations for once daily dosage forms.

Alfuzosin hydrochloride, a selective alpha adrenergic antagonist is used against benign prostatic hypertrophy (BPH)[1–3] in elderly males. The prostate gland of the patients enlarges in BPH and prevents urine flow from bladder which results in urinary retention. The treatments available are surgical removal of excess tissue or drug therapy[4]. Two classes of drugs are used, 5-alpha reductase inhibitors and alpha adrenergic antagonists. The second class includes terazosin, doxazosin, tamsulosin and alfuzosin.

Alfuzosin is freely soluble in water[5–6], and thus readily absorbed after administration. The oral absorption is significantly aided by the presence of food. The dose of immediate release alfuzosin tablet is 2.5 mg thrice daily[7–9]. Recently 10 mg once daily extended release formulation has become available in the market[10] which is more convenient for older patients[11]. Marketed alfuzosin formulation is a three layered Geomatrix tablet that requires special facilities, high cost, more time and complex operation than conventional formulations[12]. An easier directly compressible formulation was reported by Nair *et al.*[13] which is also followed in the current experiment.

As the drug is recently introduced, the data regarding the formulation and drug excipients compatibility are inadequate. Low viscosity hydroxypropylmethylcellulose (HPMC) was used by Nair *et al.*[13] to prepare controlled release alfuzosin tablet (10 mg) that sustained drug release only for 12 h. To obtain once daily dosage form, high viscosity HPMC (such as Methocel K15M) should be used which can sustain for longer period. For freely soluble drugs like alfuzosin, a large quantity of HPMC is required to control the release that ultimately results in tablets which are difficult to swallow. This problem can be resolved by using water insoluble polymer in the formulation. Therefore, directly compressible Eudragit (RS PO) was used in present study along with Methocel K15M. Similar binary mixer was reported by several workers[14–16] who used different grades of HPMC and Eudragit for preparing matrix tablets.

HPMC is widely used in matrix formulations as a release retardant polymer[17–19] that controls drug release by quickly forming a gel barrier. When a drug is formulated with gel forming hydrocolloids such as HPMC, it swells in the gastric fluid affording a prolonged gastric residence time. On the other hand, water insoluble Eudragit RS is ammoniomethacrylate copolymer (Type B) made with copolymers of acrylate and methacrylates with quarternary ammonium group. The ammonium groups are present as salts and make the polymers permeable. The objective of the study was to investigate how high viscosity HPMC and directly compressible Eudragit combination affect the dissolution rate of alfuzosin from matrices and to study the qualitative effect of fillers on drug dissolution.

## MATERIALS AND METHODS

The following materials were used in the experiment: Alfuzosin HCl BP (Standard Chem. and Pharma Co. Ltd, Taiwan), microcrystalline cellulose PH 101 (MCC; Ming Tai Chemical Co. Ltd., Taiwan), high viscosity HPMC (Methocel K15M CR, The Dow Chemical Company, USA), Eudragit RS PO (Rohm GmbH, Germany), magnesium stearate (Paul Lohman, Germany), lactose (Flowlac, Meggle GmbH, Germany) and dibasic calcium phosphate (Interpharm ltd., UK). Other chemicals used were reagent grade.

### Experimental Design:

A 3^2^ full factorial design was adopted for the experiment where two variables (X_1_, X_2_) were the amount of the two release controlling polymers as shown in Table 1. The selected responses for all possible 9 formulations were percent of drug release at 1, 6, 12 and 20 h. Methocel K15M was evaluated at 30, 40 and 50% while Eudragit RS PO was evaluated at 20, 30 and 40%.

**TABLE 1:** COMPOSITION OF TABLET FORMULATIONS

Formulation code	Weight (mg)/ Tablet					
	
	Alfuzosin HCl	Methocel K15 MCR	Eudragit RS PO	Magnesium Stearate	MCC 101	Total
F1	10	150	60	3	77	300
F2	10	90	120	3	77	300
F3	10	90	90	3	107	300
F4	10	150	90	3	47	300
F5	10	120	120	3	47	300
F6	10	150	120	3	17	300
F7	10	120	60	3	107	300
F8	10	120	90	3	77	300
F9	10	90	60	3	137	300

All batches contain 10 mg drug, 1% magnesium stearate as lubricant, and microcrystalline cellulose (MCC 101) as filler which was used to adjust the tablet weight to 300 mg.

### Preparation of Matrix tablets:

Tablets were fabricated by direct compression according to the formula given in Table 1. The amount of active ingredient and tablet weight was held constant. All ingredients except lubricant were sieved through # 40 mesh and mixed manually for 10 min. Magnesium stearate (1%) was then added after passing through # 60 mesh and the powder mixture was blended for 2 min. The tablets were compressed with B type 16 station rotary compression machine (Manesty, UK) using 10 mm diameter punches with 1.5 ton compression force.

### Physical Evaluation of Tablets:

Hardness was determined using a Monsanto hardness tester while friability was determined according to British Pharmacopoeia with a Roche friabilator (Erweka, Germany). Bulk density and tapped density of the powder blend was determined with graduated cylinders according to USP guidelines. Hausner ratio and Carr's index was determined to assess the flow property and compressibility of the powder blend[20].

### *In vitro* dissolution studies:

Dissolution studies were carried out for extended release Alfuzosin formulations using 0.01N HCl as dissolution medium[21]. The amount of drug dissolved in the medium was determined by UV spectrophotometer (Shimadzu, Japan) at 244 nm wavelength. Dissolution studies were conducted by USP method 2 at 100 rpm[21] and the temperature was maintained at 37±0.5°. As the tablets have floating tendency, metallic sinker was used to keep tablets immersed into the medium.

**Fig. 1:**
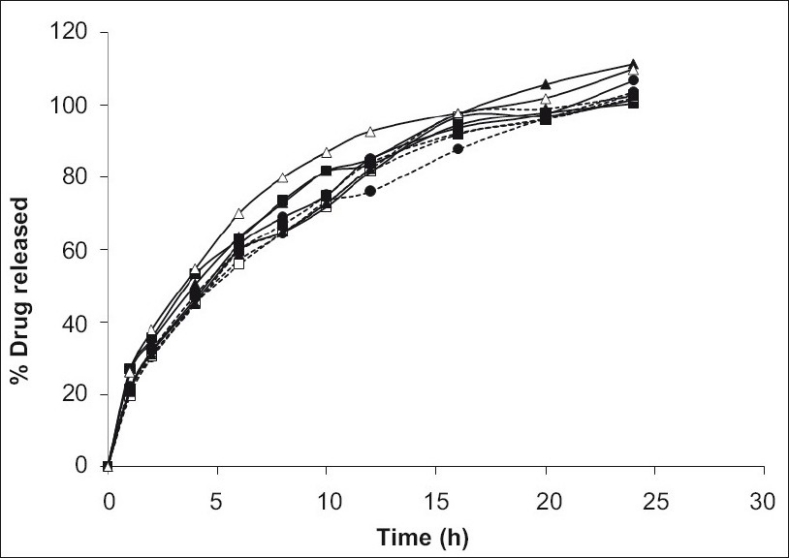
Zero order release profile of alfuzosin HCl. Cumulative percent drug release from formulations F1 (---●---), F2 (—▪—), F3 (—▲—), F4 (---□--), F5 (----▪---), F6 (---▲---), F7 (—□—), F8 (—●—), and F9 (—△—). Ratio (w/w) of Methocel and Eudragit RS PO is 150/60 (F1), 90/120 (F2), 90/90 (F3), 150/90 (F4), 120/120 (F5), 150/120 (F6), 120/60 (F7), 120/90 (F8), and 90/60 (F9). Mean of 3 runs. Error bars omitted for the sake of clarity.

This operation was continued for 24 h while samples of 5 ml were withdrawn at regular interval from the dissolution medium and replaced with fresh dissolution medium to maintain the volume constant. The samples were filtered and suitably diluted. Drug dissolved at specified time periods was plotted as mean percent release versus time (h) curve (fig. 1). This drug release profile was fitted into several mathematical models to get an insight of the release mechanism of the drug from the dosage form.

### Drug Release Kinetics:

The rate of drug release from the preparation may follow zero order kinetics, first order kinetics or Higuchi's model. To evaluate the mechanism of drug release from the preparation, data of drug release may be plotted in Korsmeyer *et al*'s equation[22], Log (M_t_/M_f_) = Log k+nLogt, which is often used to describe the drug release behavior from polymeric systems, where M_t_ is the amount of drug release at time t, M_f_ is the amount of drug release after infinite time; k is a release rate constant incorporating structural and geometric characteristics of the dosage form, n is the diffusional exponent indicative of the mechanism of drug release[23].

The log value of percent drug dissolved is plotted against log time for each formulation according to the equation. For a cylinder shaped matrix the value of n ≤ 0.45 indicates Fickian (case I) release; > 0.45 but < 0.89 for non-Fickian (anomalous) release; and > 0.89 indicates super case II type of release. Case II generally refers to the erosion of the polymer and anomalous transport (Non-Fickian) refers to a combination of both diffusion and erosion controlled drug release[24].

Mean Dissolution Time (MDT) can be calculated from dissolution data according to Mockel and Lippold[25] equation to characterize the drug release rate from the dosage form and the retarding efficiency of the polymer. MDT value can be calculated from dissolution data using the equation MDT = (n/n+1)k^-1/n^, where n is the release exponent and k is release rate constant.

A higher value of MDT indicates a higher drug retaining ability of the polymer and vice-versa. Similarity factor (f_2_) is the measurement of similarity of two different dissolution curves. When f_2_ value is greater than 50, the curves are similar. The value is determined by the equation f_2_ = 50log[{1+(1/n) Σ(R_t_-T_t_)^2^}^−0.5^.100], where n is the number of dissolution sample times, and R_t_ and T_t_ are the individual percentages dissolved at each time point t for the reference and test dissolution profiles respectively[26].

### Statistical analysis:

The response surface graphs and multiple regression analysis were carried out by Design Expert 7.0 (Stat-Ease Inc., Minneapolis, Minnesota) software at 5% significance level.

### Stability study:

Stability study of selected formulations was tested according to International Conference of Harmonization guideline. The tablets were stored in Alu-Alu blister for 3 mo in stability chamber (Memmert, Germany) at 40°/75% RH. After 3 mo, tablets were tested for drug content and *in vitro* dissolution.

## RESULTS AND DISCUSSION

The weight variation of the tablets ranged between 0.13-3.0% (1.25±0.39%), thickness between 4.2-4.5 mm (4.35±0.03 mm) and hardness was within 0.5-2.15 kg/cm^2^ (1.14±0.18 kg/cm^2^) as shown in Table 2. The hardness of the tablets increased proportionally with the amount of Methocel K15M due the binding property of HPMC. The weight variation and friability of the batches complied with British Pharmacopoeia. The tablets were prepared by direct compression method, therefore the particle size and flow property of the powder blend should be in acceptable range. The particle size was on an average 420 μm and flow property was determined by Hausner ratio (1.37-1.42) and Carr's Index (27.14%-29.58%). The data proved that the flow properties and compressibility of blends were satisfactory[20]. Thus all the physical parameters of different batches were within control.

**TABLE 2:** PHYSICAL CHARACTERISTICS OF TABLETS

Code	Weight variation %	Hardness (Kg/cm^2^)	Friability %	Bulk density (loose) g/cm^2^	Bulk density (tapped) g/cm^2^	Carr's index	Hausner ratio
F1	0.25	2.15	0.01	0.50	0.70	28.57	1.40
F2	3.00	1.00	0.01	0.51	0.71	28.17	1.39
F3	3.00	0.50	0.01	0.51	0.71	28.17	1.39
F4	0.16	2.00	0.01	0.52	0.72	27.78	1.38
F5	1.28	1.00	0.01	0.51	0.70	27.14	1.37
F6	0.13	1.00	0.01	0.50	0.70	28.57	1.40
F7	2.00	1.00	0.01	0.50	0.70	28.57	1.40
F8	1.00	0.80	0.01	0.50	0.71	29.58	1.42
F9	0.40	0.80	0.01	0.51	0.72	29.17	1.41

Mean values of all physical properties tested are shown in the table.

About 25% of drug was released within 1^st^ h (t_25%_ = 1.11±0.23 h, mean±SD) of *in vitro* dissolution. It took 4-5 h to release about 50% of drug (t_50%_ = 4.59±0.6 h, mean±SD) and 75% drug released within 10 h (t_75%_ =10.56±0.94 h, mean±S.D.) from all formulations (Table 3, fig. 1). The highest release retardant formulations were 150/60,150/90 and 120/60 Methocel-Eudragit (w/w) ratio as determined by their MDT values. The gradual increase of the acrylic polymer did not affect the drug release rate as indicated by f_2_ calculation. Among Methocel-Eudragit w/w ratio of 90/60, 90/90 and 90/120, the first formulation was taken as reference (R_t_). The data indicates up to 40% Eudragit is not sufficient for reducing release of freely soluble drug like alfuzosin. On the other hand, when the Eudragit quantity was kept constant and HPMC level was increased, the drug release continued to fall. For instance, in Methocel-Eudragit w/w 90/60, 120/60 and 150/60 ratios the mean drug release after 12 h was 92.62, 81.59, and 76.12%, respectively.

**TABLE 3:** RELEASE KINETICS OF TABLETS

Kinetic parameters	Formulation code								
	
	F1	F2	F3	F4	F5	F6	F7	F8	F9
Zero order									
r^2^	0.89	0.81	0.87	0.88	0.86	0.87	0.86	0.87	0.80
K_0_	3.85	3.67	4.13	3.92	3.86	3.99	3.90	3.99	3.95
First order									
r^2^	0.95	0.99	0.96	0.97	0.97	0.94	0.96	0.98	0.98
K_1_	0.08	0.08	0.09	0.08	0.08	0.09	0.08	0.08	0.09
Higuchi									
r^2^	0.99	0.97	0.99	0.99	0.99	0.99	0.99	0.99	0.97
K_H_	21.45	21.15	23.20	21.91	21.76	22.36	21.92	22.38	22.81
Korsmeyer									
r^2^	0.99	0.98	0.99	0.99	0.99	0.99	0.99	0.99	0.98
n	0.49	0.43	0.47	0.52	0.49	0.52	0.50	0.50	0.45
K	0.23	0.28	0.26	0.21	0.23	0.22	0.22	0.23	0.28
MDT	6.90	5.80	5.62	6.88	6.60	6.29	6.8	6.30	5.25
t_25%_	1.18	0.77	0.92	1.40	1.18	1.28	1.29	1.18	0.78
t_50%_	4.88	3.85	4.02	5.30	4.88	4.85	5.16	4.73	3.63
t_75%_	11.16	9.89	9.53	11.56	11.16	10.57	11.62	10.63	8.93
t_90%_	16.19	15.11	14.04	16.42	16.19	15.02	16.73	15.31	13.39

K_0_ K_1_,K_H_ and K are the rate constants for zero order, first order, Higuchi and Korsmeyer respectively. n is the diffusion exponent.

**Fig. 2:**
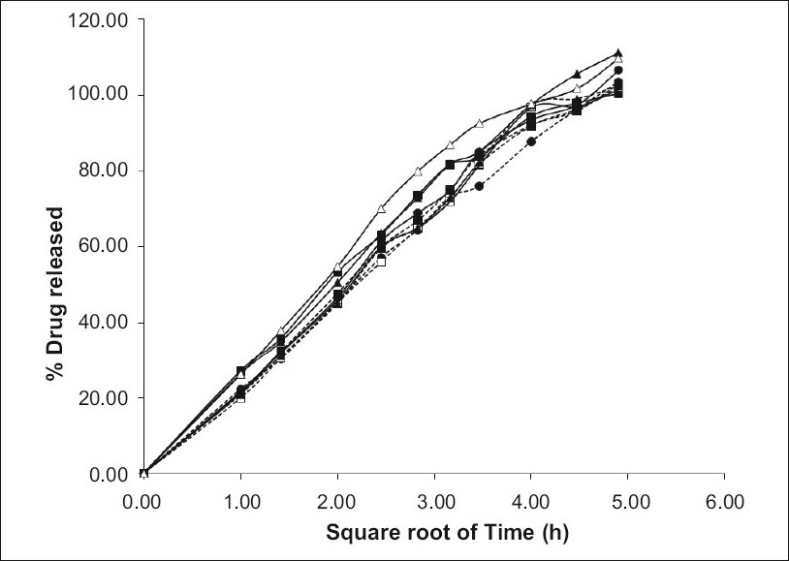
Higuchi release profile of Alfuzosin HCl. Higuchi drug release from the formulations F1 (---●---), F2 (—▪—), F3 (—▲—), F4 (---□---), F5 (----▪---), F6 (---▲---), F7 (—□—), F8 (—●—), and F9 (—△—). Ratio (w/w) of Methocel and Eudragit RS PO is 150/60 (F1), 90/120 (F2), 90/90 (F3), 150/90 (F4), 120/120 (F5), 150/120 (F6), 120/60 (F7), 120/90 (F8) and 90/60 (F9). (n=3)

**Fig. 3:**
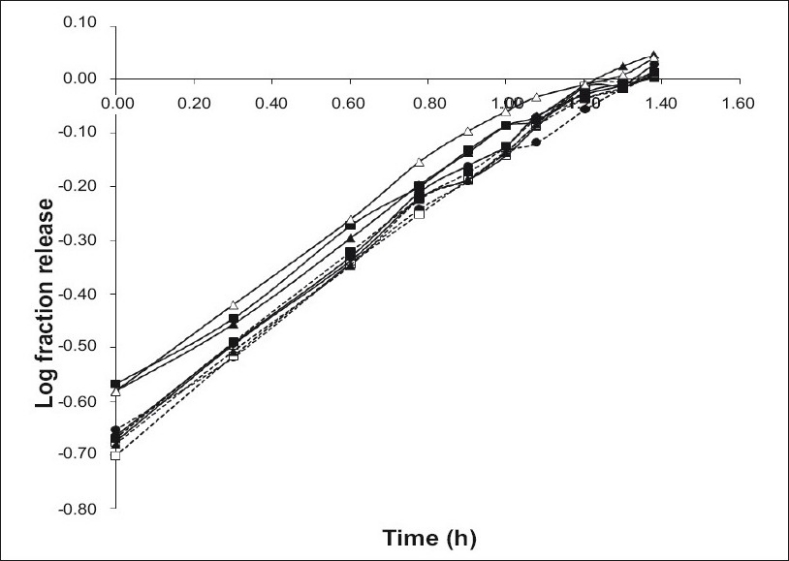
Korsmeyer release profile of Alfuzosin HCl. Korsmeyer release profile of formulations F1 (---●---), F2 (—▪—), F3 (—▲—), F4 (---□--), F5 (----▪---), F6 (---▲---), F7 (—□—), F8 (—●—), and F9 (—△—). Ratio (w/w) of Methocel and Eudragit RS PO is 150/60(F1), 90/120 (F2), 90/90 (F3), 150/90 (F4), 120/120 (F5), 150/120 (F6), 120/60 (F7), 120/90 (F8) and 90/60 (F9). (n=3)

All the tablets showed good fit for Higuchi (r^2^=0.97-0.99) and Korsmeyer (r^2^=0.98-0.99) kinetic models (Table 3; figs. 2 and 3). From Higuchi model it is evident that alfuzosin is released by diffusion process from the matrices. HPMC controls the release of soluble drugs by diffusion process and poorly soluble drugs by both diffusion and erosion mechanism[27–29]. This diffusion is probably due to the presence of gel barrier of HPMC. The diffusion exponent (n) of Korsmeyer model ranged from 0.43-0.52 indicating anomalous or non-Fickian transport. Therefore both diffusion and erosion mechanisms play role in alfuzosin release from Methocel-Eudragit matrix.

**Fig. 4:**
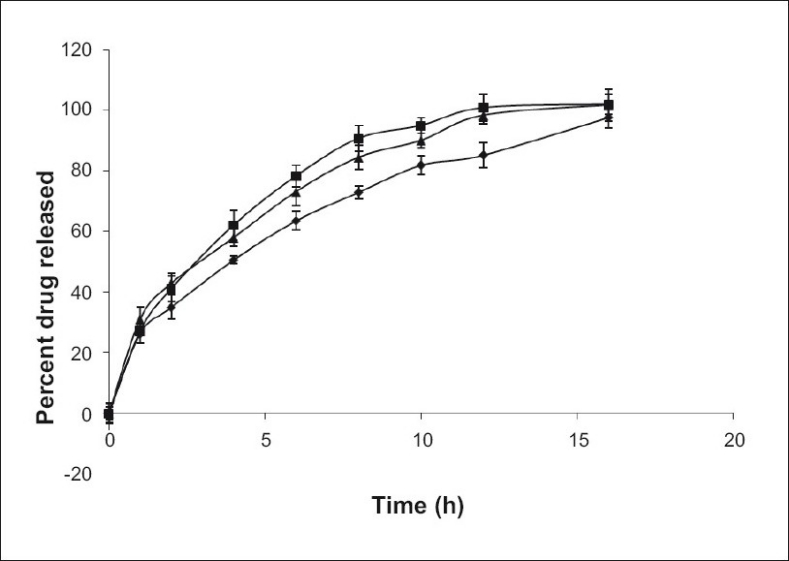
Effect of filler on Alfuzosin matrix tablets prepared with 30% HPMC and 30% Eudragit RS PO. Microcrystalline cellulose (—♦—) was replaced by lactose (—▪—) and dibasic calcium phosphate (—▲—). Mean±SD, n=3

The tablets in the experiment were primarily prepared with microcrystalline cellulose (MCC) as filler. The effect of other fillers, such as directly compressible lactose and dibasic calcium phosphate dihydrate (DCP) on release of alfuzosin hydrochloride was also investigated. Lactose can act as a channeling agent due to its solubility while DCP can prevent water infiltration for its insoluble nature. Equal amounts of lactose or DCP replaced MCC in the formulations. Lactose containing tablets released alfuzosin faster than MCC and DCP as the release profiles of corresponding formulations were not similar (f_2_<50). Tablets containing DCP released slightly more drug than MCC containing formulation (fig. 4) as it tends to dissolve slowly in acidic media but the release profiles were not significantly different (f_2_>50).

**Fig. 5:**
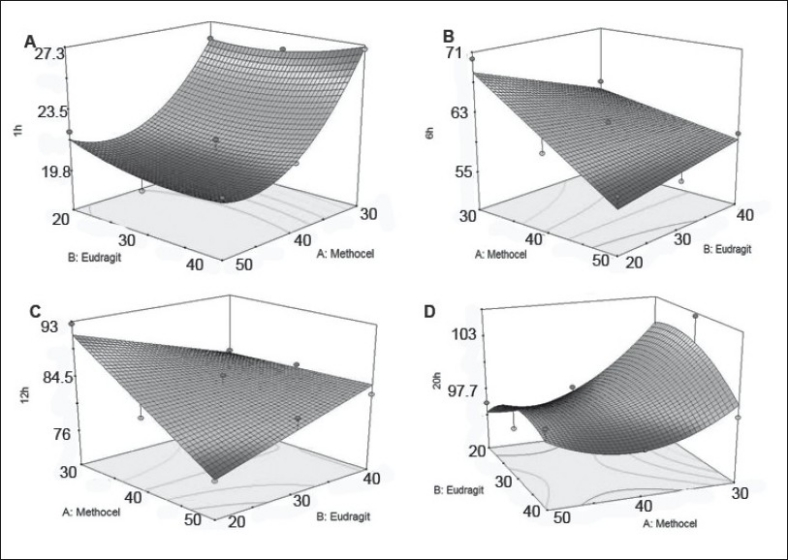
Response surface plots showing effect of polymer content on drug release. A. Drug release at 1h, B. drug release at 6h, C. drug release at 12h, and D. drug release at 20h

The drug release percentages at 1, 6, 12 and 20 h were selected as response variable (Table 4). These time periods are selected to detect any initial burst effect, time for 50% and 90% drug release. The equations of all responses (Table 5) represent the quantitative effect of independent variables upon the responses. A positive sign indicates a synergistic effect while a negative sign indicates antagonistic effect upon the responses[30]. b_0_ is the arithmetic mean response of the 9 runs and b_i_ is the estimated coefficient for X_i_ (Table 5). It was found that Methocel (X_2_) was responsible for reducing drug release significantly (p<0.05) at 1, 6 and 12 h. Positive interaction between Methocel and Eudragit was found during 6 and 12 h (Table 5). Response surfaces depicting the effect of the casual factors on each response variable are presented in fig. 5.

**TABLE 4:** THE CASUAL FACTOR AND RESPONSES OF MODEL FORMULATIONS

Formulation code	X_1_	X_2_	Y_1_	Y_2_	Y_3_	Y_4_
F1	-1	1	22.23	57.20	76.12	96.29
F2	1	-1	27.05	63.13	83.69	97.90
F3	0	-1	26.25	63.56	85.21	105.66
F4	0	1	19.88	56.00	81.90	96.26
F5	1	0	21.42	59.94	84.12	95.85
F6	1	1	20.88	59.67	82.46	99.00
F7	-1	0	21.09	59.99	81.59	97.00
F8	0	0	21.65	61.50	85.11	97.54
F9	-1	-1	26.18	70.14	92.62	101.85

X_1_ and X_2_ are the amount of Eudragit RS and Methocel K15MCR respectively. The formulations are according to Table 1.Y: responses, the release percent at 1 h (Y_1_), 6 h (Y_2_), 12 h (Y_3_) and 20 h (Y_4_). Coded value 0 represents 30% for X1 and 40% for X_2_, coded value -1 represents 20% for X1 and 30% for X_2_, and coded value 1 represents 40% for X_1_ and 50% for X_2_.

**TABLE 5:** REGRESION EQUATION FOR EACH RESPONSE VARIABLE DETERMINED BY MULTIPLE REGRESION ANALYSIS

Regression coefficient	Independent variables	Y_1_	Y_2_	Y_3_	Y_4_
b_0_		21.02111	61.23667	83.64667	-
b_1_	X_1_	-	-	-	-
b_2_	X_2_	-2.74833	-3.99333	-3.50667	-
b_12_	X_1_X_2_	-	2.37	3.8175	-
b_11_	X_1_X_1_	-	-	-	-
b_22_	X_2_X_2_	2.358333	-	-	

Insignificant values (p>0.05) are not shown on regression equation.

**Fig. 6:**
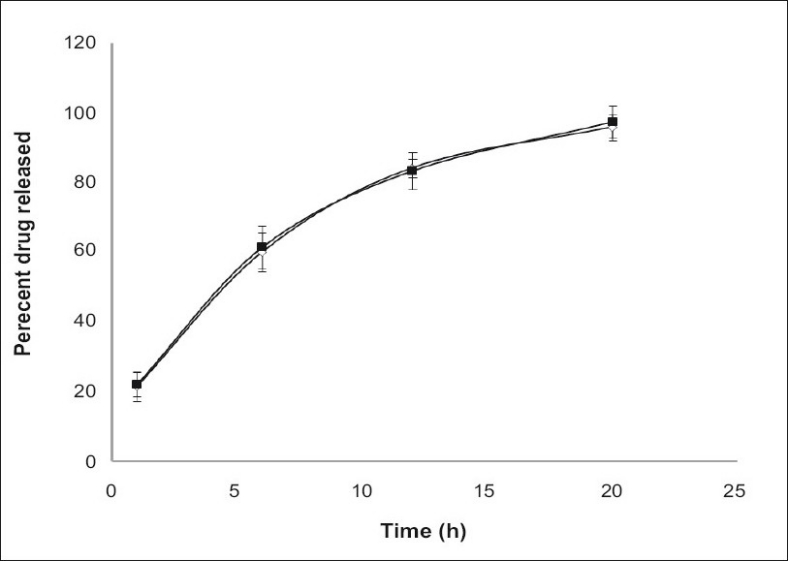
*In vitro* dissolution of initial and 3 months stability studies of Alfuzosin matrix tablets. *In vitro* dissolution of initial (—◇—) and 3 months stability studies (—▪—) of alfuzosin matrix tablets containing 40% w/w Methocel K15 M and 40% w/w Eudragit RS PO. Mean± SD, n=3.

The physical, chemical and *in vitro* release of tablets kept at 40°/75% RH were studied according to previously mentioned methods revealed no significant change from initial values (fig. 6) which indicates the formulations were stable. Combination of two different polymers yielded tablets of acceptable physical characteristics and chemical stability. When used in combination, the role of HPMC in sustaining drug release is more prominent than Eudragit polymer and less amount of these polymers are required if MCC is used as a filler. In conclusion, stable extended release matrix tablets of alfuzosin can be prepared by adjusting the ratio of binary polymers and by selecting the suitable filler which can control drug release up to 20 h.
